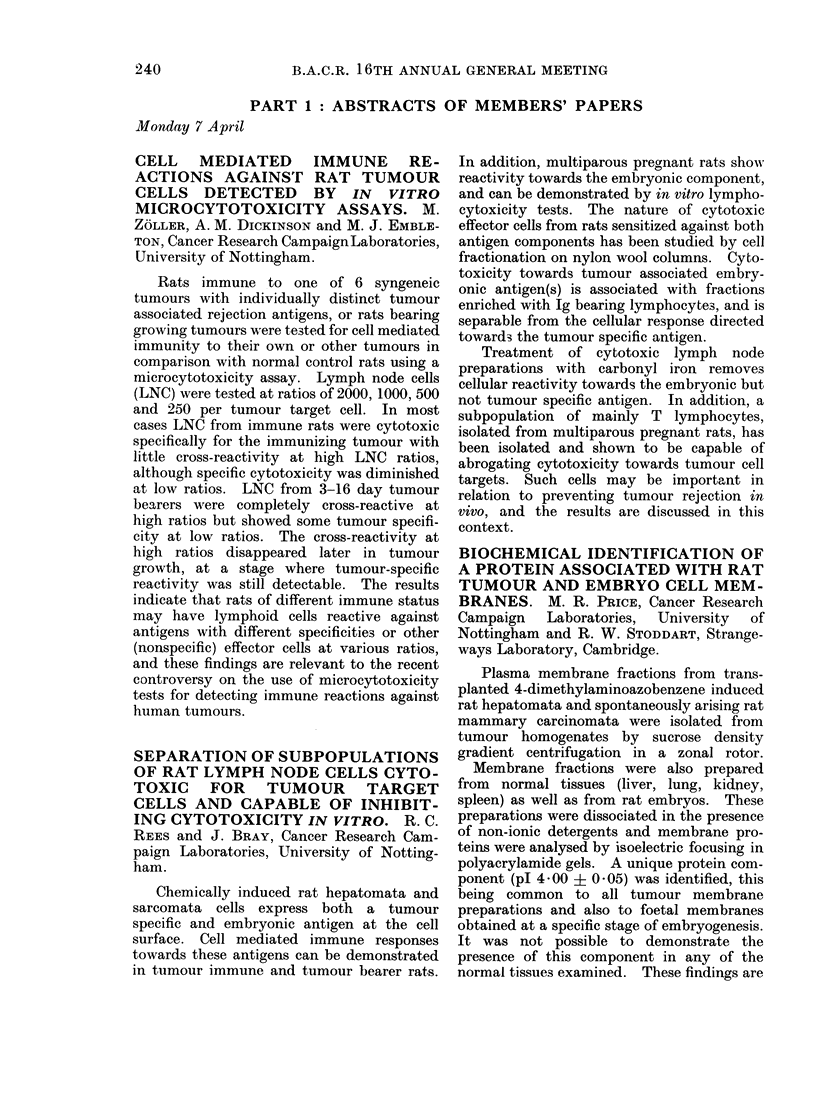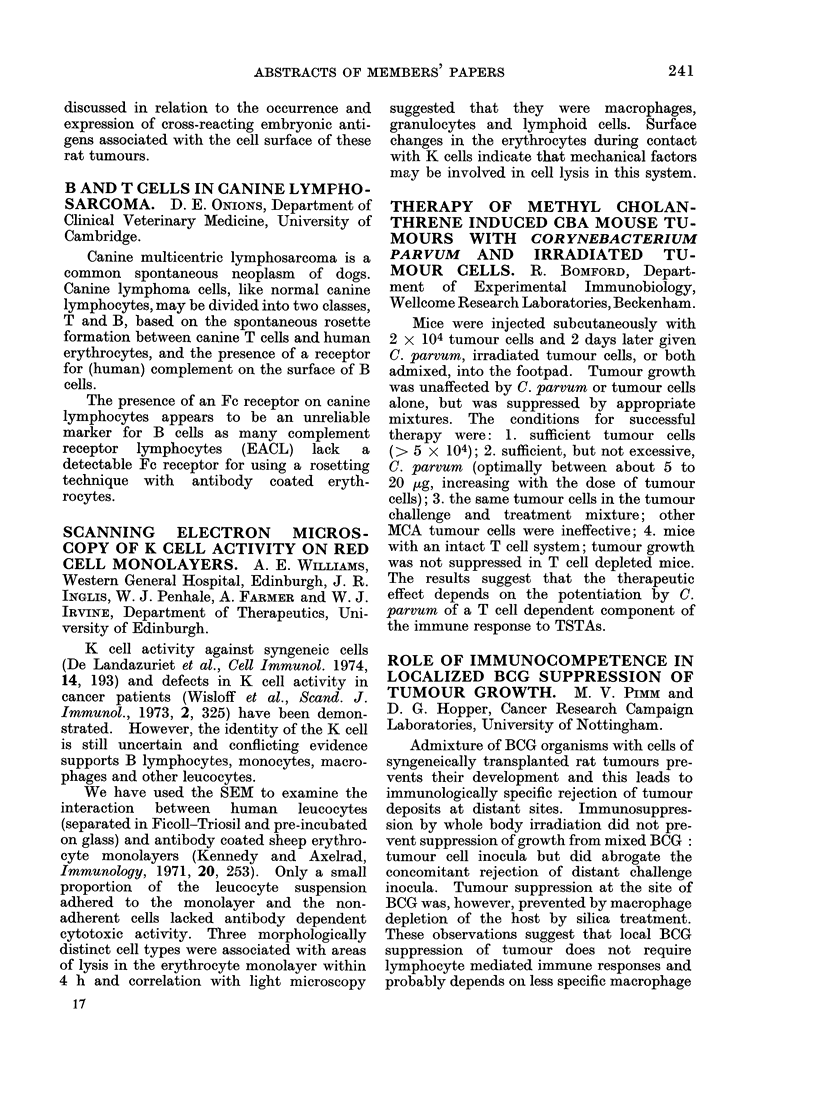# Proceedings: Biochemical identification of a protein associated with rat tumour and embryo cell membranes.

**DOI:** 10.1038/bjc.1975.156

**Published:** 1975-08

**Authors:** M. R. Price, R. W. Stoddart


### PART 1 : ABSTRACTS OF MEMBERS' PAPERS

### BIOCHEMICAL IDENTIFICATION OF A PROTEIN ASSOCIATED WITH RAT TUMOUR AND EMBRYO CELL MEMBRANES.

M. R. Price, Cancer Research Campaign Laboratories, University of Nottingham and R. W. Stoddart, Strangeways Laboratory, Cambridge.

Plasma membrane fractions from transplanted 4-dimethylaminoazobenzene induced rat hepatomata and spontaneously arising rat mammary carcinomata were isolated from tumour homogenates by sucrose density gradient centrifugation in a zonal rotor. Membrane fractions were also prepared from normal tissues (liver, lung, kidney, spleen) as well as from rat embryos. These preparations were dissociated in the presence of non-ionic detergents and membrane proteins were analysed by isoelectric focusing in polyacrylamide gels. A unique protein component (pI $4{\cdot}00 \pm 0{\cdot}05$) was identified, this being common to all tumour membrane preparations and also to foetal membranes obtained at a specific stage of embryogenesis. It was not possible to demonstrate the presence of this component in any of the normal tissues examined. These findings are discussed in relation to the occurrence and expression of cross-reacting embryonic antigens associated with the cell surface of these rat tumours.